# Breaking Bad News in the Emergency Department

**DOI:** 10.21980/J81W7H

**Published:** 2022-04-15

**Authors:** Susan Siraco, Cindy Bitter, Tina Chen

**Affiliations:** * Saint Louis University School of Medicine, Saint Louis, MO; ^ Saint Louis University, Division of Emergency Medicine, Saint Louis, MO

## Abstract

**Audience:**

The primary audience for this simulation is emergency medicine (EM) residents, but this curriculum could also be used for EM-bound medical students.

**Introduction:**

Breaking bad news is a difficult but necessary skill for EM physicians. Bad news can range from informing family that a patient is in the emergency department (ED), to shared decision making regarding a life-threatening situation, to family notification of patient death.^1^ Although there are many structured approaches to death notification and breaking bad news, such as GRIEV_ING^2^ and SPIKES,^3^ EM physicians often lack confidence in their ability to effectively communicate bad news.^1^,^4^–^6^ Goals of care discussions and shared decision making become especially complex in the ED environment because critically ill patients often arrive without advanced directives, lack pre-existing rapport with the EM physician, and may require rapid engagement with surrogate decision-makers on emergent interventions.^7^ This simulation curriculum was developed to provide EM trainees with a psychologically safe environment to practice effective communication in breaking bad news, incorporating clinical scenarios commonly encountered in the ED.

**Educational Objectives:**

At the conclusion of these two simulation cases, learners will be able to 1) recognize signs of poor prognosis requiring emergent family notification, 2) take practical steps to contact family using available resources and personnel, 3) establish goals of care through effective family discussion, 4) use a structured approach, such as GRIEV_ING, to deliver bad news to patients’ families, and 5) name the advantages of family-witnessed resuscitation.

**Educational Methods:**

This curriculum consists of two simulation cases. Prior to the simulation, learners were assigned pre-reading on the GRIEV_ING approach to death notification, and how this approach could translate into breaking bad news in the ED. Although we chose to implement GRIEV_ING at our institution, other structured approaches (such as SPIKES) are reasonable as well. Each simulation case was conducted using a high-fidelity mannequin capable of intubation, respiratory examination findings such as abnormal breath sounds, and dynamic vital sign changes. Both cases required a standardized patient or other case confederate. Following each case, the learners underwent a debriefing session discussing how to break bad news in a high-pressure, time-sensitive ED environment. This case was designed as a high-fidelity simulation with a standardized patient, but it can be adapted to a low-fidelity simulation with a standardized patient.

**Research Methods:**

Learners filled out a survey before and after the simulation describing their confidence in establishing goals of care with patients and surrogates, notifying family members of bad news in the ED, and their use of a consistent approach to breaking bad news. Scores were analyzed using the related-samples Wilcoxon signed rank test.

**Results:**

Learners exhibited improvement on all surveyed items, with statistically significant improvement on the survey item asking about their confidence in implementing a consistent approach to breaking bad news. Qualitative feedback was positive, with learners consistently endorsing the value of practicing difficult conversations in a simulated environment. First- and second-year residents appeared to benefit from the cases more strongly than senior residents.

**Discussion:**

These cases provided a safe environment for learners to practice a structured approach to breaking bad news. Learners tended to aggressively resuscitate the elderly septic patient and perform invasive procedures, such as intubation and mechanical ventilation, prior to contacting family or establishing goals of care, which generated good discussion points during debriefing.

**Topics:**

Simulation, breaking bad news, goals of care discussion, death notification, sepsis, cardiac arrest, family witnessed resuscitation.

## USER GUIDE

**Table:** 

List of Resources:	
Abstract	1
User Guide	3
Case 1: Instructor Materials	6
Case 1: Standardized Patient Briefing Materials	14
Case 1: Operator Materials	18
Case 1: Debriefing and Evaluation Pearls	20
Case 1: Simulation Assessment	23
Case 2: Instructor Materials	28
Case 2: Standardized Patient Briefing Materials	35
Case 2: Operator Materials	39
Case 2: Debriefing and Evaluation Pearls	41
Case 2: Simulation Assessment	43

**Learner Audience:**

Medical students, interns, junior residents, senior residents

**Time Required for Implementation:**

**Instructor Preparation:** 30 minutes to set up equipment and rooms the day of the simulation. 1–2 hours to recruit and train the standardized patient or case confederate and review simulation materials in advance of the simulation.

**Time for case:** 20 minutes per case.

**Time for debriefing:** 30 minutes per case.

**Recommended Number of Learners per Instructor:**

2–4 learners per instructor per case. We recommend keeping the groups small.

**Topics:**

Simulation, breaking bad news, goals of care discussion, death notification, sepsis, cardiac arrest, family witnessed resuscitation.

**Objectives:**

At the conclusion of this simulation curriculum, learners will be able to:

1. Recognize signs of poor prognosis requiring emergent family notification
2. Take practical steps to contact family using available resources and personnel
3. Establish goals of care through effective family discussion
4. Use a structured approach, such as GRIEV_ING, to deliver bad news to patient’s families
5. Name the advantages of family-witnessed resuscitation.

### Linked objectives and methods

EM physicians must be capable of empathetic and timely communication with family members in the fast-paced, high-stakes environment of the ED. This is especially important for critically ill patients with expected poor prognosis who may require time-sensitive interventions or compassionate palliation, depending on the patient’s values and wishes.

These two simulation cases highlight the challenges of sensitive family discussions in the ED environment, allowing learners to practice difficult aspects of breaking bad news in a high-fidelity but psychologically safe simulation environment. Each case emphasizes early recognition of poor patient prognosis, timely family notification, effective elicitation of patient values for shared decision-making, and use of compassionate but unambiguous language in family communications. Additionally, one of the two cases (“Family Witnessed Resuscitation”) provides learners with the opportunity to offer family witnessed resuscitation, a practice which has been linked to better bereavement adjustment for surviving family. The learner-responsible reading and debriefing materials highlight the benefits of using a structured communication framework, GRIEV_ING, to comprehensively guide learners through the multifaceted challenges of breaking bad news.

We chose to employ GRIEV_ING in our curriculum because it has previously been shown to be an effective teaching and assessment tool in death notification skills among EM residents.^2^ However, other structured approaches, such as SPIKES, may also be appropriate, depending on facilitator judgment, institutional norms, and previous learner experiences.

### Recommended pre-reading for instructor

1. Hobgood C. The Educational Intervention “GRIEV_ING” Improves the Death Notification Skills of Residents. *Academic Emergency Medicine*. 2005;12(4):296–301. doi:10.1197/j.aem.2004.12.008
2. Ouchi K, George N, Schuur JD, et al. Goals-of-Care Conversations for Older Adults with Serious Illness in the Emergency Department: Challenges and Opportunities. *Annals of Emergency Medicine*. 2019;74(2):276–284. doi:10.1016/j.annemergmed.2019.01.003
3. Jabre P, Belpomme V, Azoulay E, et al. Family Presence during Cardiopulmonary Resuscitation. *N Engl J Med*. 2013;368(11):1008–1018. doi:10.1056/NEJMoa1203366

### Learner responsible content

- https://www.nuemblog.com/blog/death-notification
- If the curriculum facilitator chooses to utilize another communication strategy for breaking bad news, such

### Results and tips for successful implementation

This curriculum was deployed with a total of thirteen EM residents in various levels of training. Learners filled out a survey before and after the simulation describing their confidence in establishing goals of care with patients and surrogates, notifying family members of bad news in the ED, and their use of a consistent approach to breaking bad news.

**Figure 1:**
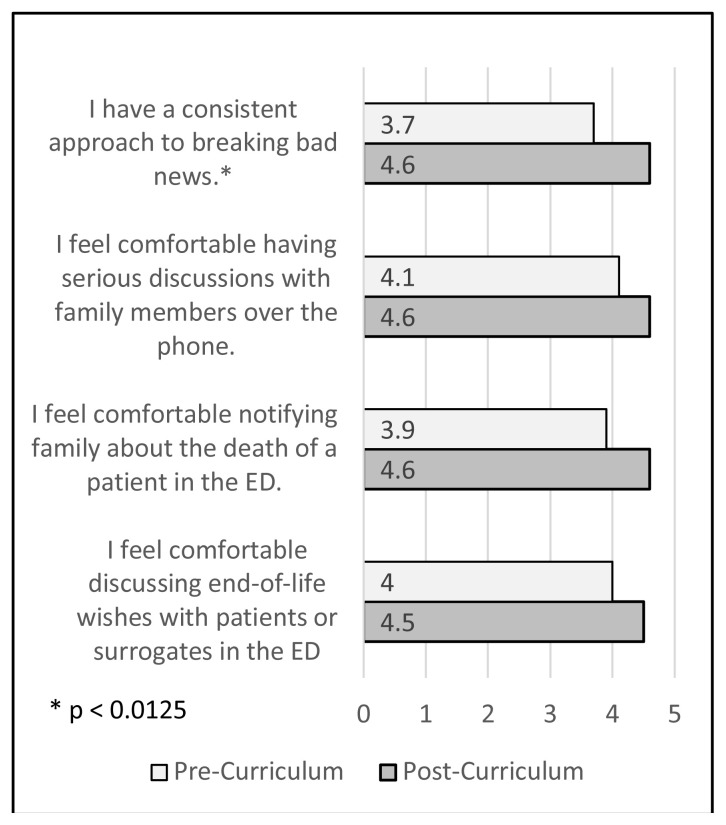
Pre- and Post-Curriculum Survey Responses

Learners were surveyed on their comfort managing end-of-life discussions before and after the curriculum, on a Likert scale where 1 = strongly disagree, 2 = disagree, 3 = neither agree nor disagree, 4 = agree, 5 = strongly agree. Pre- and post-curriculum scores for each question in the evaluation were evaluated using the related-samples Wilcoxon signed rank test, with the p-value adjusted using the Bonferroni correction for multiple comparisons. Learners indicated improved confidence on all surveyed items, with statistically significant improvement on the survey item asking about their confidence in implementing a consistent approach to breaking bad news (Figure 1).

Qualitative feedback on the session was positive, with comments such as, “it is always good to review how to do this effectively, so we don’t fumble around in the real thing,” and “very important, informative, and greatly appreciated. Strongly encourage continued use of these cases.” We noticed during implementation of the simulation cases that learners, regardless of post-graduate year level, tended to perform invasive interventions such as intubation and mechanical ventilation prior to contacting the power of attorney (POA) in the first simulation case (“Connecting Remotely”). The case was specifically written such that the patient’s hypoxia and hypotension improved with less invasive interventions, such as intravenous fluids and supplemental oxygen, though her vital signs did not normalize; upon review by other EM clinical faculty, there was consensus that the patient’s condition would permit time to contact a family member. This served as an effective discussion point during the debrief on the value of establishing goals of care prior to invasive interventions. Additionally, many junior learners were unaware of family-witnessed resuscitation.

### Associated Materials

- Training materials for the standardized patient or case confederate.

## Supplementary Information



## Figures and Tables

**Table:** 

#1	Lab results demonstrating leukocytosis and lactic acidosis
#2	EKG with sinus tachycardia
#3	Chest X-ray with multifocal pneumonia

**Table:** 

**Age**: 80	**Sex**: Female	**Method of Transportation**: EMS

**Table:** 

**Vitals:**	Heart Rate (HR) 135	Blood Pressure (BP) 80/40	Respiratory Rate (RR) 32
	Temperature (T) 101.8°F	Oxygen Saturation (O_2_Sat) 80% on room air	

**Table:** 

*Complete blood count (CBC)*	
White blood count (WBC)	16.2 × 1000/mm^3^ (H)
Hemoglobin (Hgb)	11.2 g/dL
Hematocrit (HCT)	34.4%
Platelet (Plt)	400 × 1000/mm^3^
Segs:	88%
Lymphs:	6%
Monos:	4%
Eos:	2%

**Table:** 

*Basic metabolic panel (BMP)*	
Sodium	132 mEq/L (L)
Potassium	3.7 mEq/L
Chloride	98 mEq/L (L)
Bicarbonate (HCO_3_)	11 mEq/L (L)
Blood Urea Nitrogen (BUN)	34 mg/dL (H)
Creatinine (Cr)	2.4 mg/dL (H)
Glucose	95 mg/dL

**Table:** 

*Lactate*	8.7 mmol/L (H)

**Figure:**
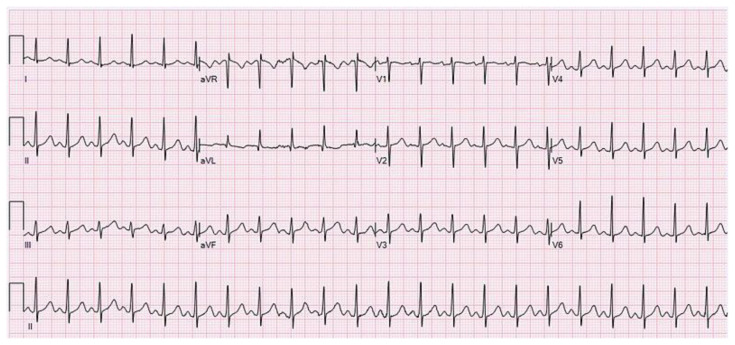


**Figure:**
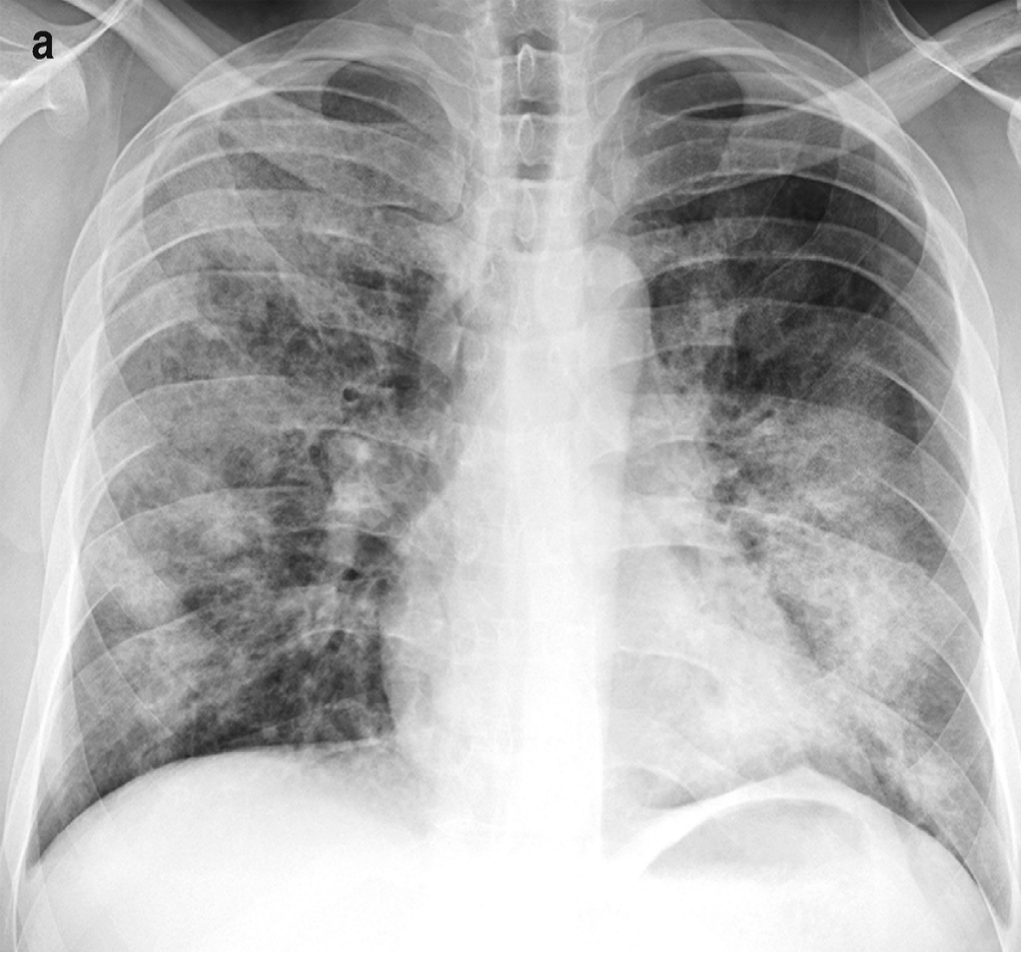


**Table:** 

*Learner Actions*	*Suggested Statements*
Learner contacts you by phone.	Introduce yourself as the patient’s significant other, sibling, or child (depending on your age and your preference).
Learner describes the patient’s severe pneumonia.	Ask clarifying questions, especially if the learner uses too much technical terminology. Possible questions could include: - “You used a word, sepsis. What does that mean?” - “What does it mean to be intubated?”
Learner communicates the patient’s prognosis and possible management options. Maximal medical management may include putting the patient on a ventilator, administering antibiotics, placing a central line for pressors, and intensive care unit admission.	Ask clarifying questions, especially if the learner is vague or imprecise. Possible questions could include: - “Do you think that she will live through this illness, doctor?” - “I want you to be honest with me – do you think she is dying?” - “How long do you think she would be on a ventilator, if we chose to do that?” - “How long do you think she would be in the hospital?” - “When do you think she would make it home?”
Learner elicits the patient’s wishes for her life and death.	Do your best to advocate for the patient: - “Well, she really values living at home. She takes care of all her own cooking and cleaning. She hated not being able to take care of herself the last time she was hospitalized.” - “She always told me to let her go if she was going to be too sick to live by herself at home.” - “She had a hard time recovering from her hip surgery. She spent more than a month in rehab. She said that she never wanted to go through that again.” - “I know she’s been working on paperwork with her doctor to avoid being put on a ventilator or having chest compressions.”
Learner outlines a care plan that accounts for the patient’s wishes.	Continue to advocate for the patient and provide positive reinforcement to the learner: - “Yes, doctor, that sounds like what she would want.” - “I appreciate you taking care of her. You’ve been very helpful.”

**Table:** 

*Learner Error*	*Suggested Statements*
Learner fails to reach out to you altogether, and proceeds with placing the patient on a ventilator.	Call the learner at the end of the case and angrily tell them that the patient would not have wanted this outcome: - “I can’t believe you didn’t call me. Isn’t my number in the chart?” - “She would never want to be on a ventilator. I can’t believe you did this to her.”
Learner does not exhibit an appropriate degree of empathy.	Attempt to slow the learner down and give them an opportunity to self-correct. Communicate your feelings to the learner, especially if they are offensive: - “I’m sorry, doctor. This is a lot to process. Can I have a moment?” - “I know she’s old, but that doesn’t mean she deserves to die.”
Learner uses too much jargon or technical terminology.	Ask clarifying questions about confusing terminology.
Learner is vague or imprecise.	Encourage clear and unambiguous language: - “What do you mean, doctor? Is she sick or not?” - “What do you mean by ‘let things take their natural course’?”
Learner does not ask about the patient’s wishes, instead framing the discussion as a series of recommendations (eg, “I plan to put your mom on a ventilator to support her breathing.”)	Attempt to slow the learner down and give them an opportunity to self-correct: - “Wait a minute, that sounds a lot like life support. She wouldn’t want that.” - “I know she doesn’t want to die, but I know that she wanted to be capable of enjoying her life.”

**Table:** SIMULATION EVENTS TABLE

Minute (state)	Participant action/ trigger	Patient status (simulator response) & operator prompts	Monitor display (vital signs)
0:00 (Base-line)	The learners take EMS report and begin patient assessment.	If learners attempt to communicate with the patient, the patient will make confused responses with no coherent speech.	T 101.8°F HR 135 BP 80/45 RR 32 O2 80% RA
02:00	Learners begin resuscitative measures, establish IV access, and obtain labs/imaging.	Learners should administer IV fluids and supplemental oxygen. If they do not do so, nursing will prompt, “The patient’s blood pressure seems very low, doctor,” or, “The patient looks quite cyanotic, doctor.” The patient’s blood pressure will improve with 1–2 L of fluid. The oxygen saturation will improve with a non-rebreather mask but fail to adequately respond to other modes of supplemental oxygen.	T 101.8°F HR 105 BP 100/70 RR 32 O2 94% NRM
10:00	Learners recognize the need for mechanical intubation and ventilation.	At this point in the case, learners may choose one of two major branch points: (1) *Learners may recognize the need to establish goals of care*. If they ask for advanced directives or family contact information, they will be provided with the phone number for the patient’s POA, to be played by an SP or case confederate. The POA will relay that the patient recently had a prolonged hospitalization, and that the patient “never wanted to go through that again.” They have begun to file “do not resuscitate/do not intubate” paperwork. Based on this discussion, learners may mutually agree with the POA that the patient would desire comfort care or de-escalation of care. (2) *Alternatively, learners may proceed with intubation without establishing goals of care*. If that is the case, after intubation, the learners will get an upset phone call from the patient’s POA stating that this was not in alignment with the patient’s wishes.	Not intubated: T 101.8°F HR 105 BP 100/70 RR 32 O2 94% NRM Intubated: T 101.8°F HR 105 BP 100/70 RR (vent) SpO2 100%
(Case Completion)	Learners admit the patient	The appropriate inpatient setting may be a medical ICU bed or a floor bed, depending on whether the POA was contacted and goals of care were discussed.	

**Table 2:** The GRIEV_ING Mnemonic Source: Hobgood *et al*, 2004

**G**	**Gather**	Gather the family; ensure that all members are present.
**R**	**Resources**	Call for support resources available to assist the family with their grief, ie, chaplain services, ministers, family, and friends.
**I**	**Identify**	Identify yourself, identify the deceased or injured patient by *name*, and identify the state of knowledge of the family relative to the events of the day.
**E**	**Educate**	Briefly educate the family as to the events that have occurred in the emergency department; educate them about the state of their loved one.
**V**	**Verify**	Verify that their family member has died. *Be clear!* Use the words “dead” or “died.”
**__**	**Space**	Give the family personal space and time for an emotional moment; allow the family time to absorb the information.
**I**	**Inquire**	Ask if there are any questions and answer them all.
**N**	**Nuts and Bolts**	Inquire about organ donation, funeral services, and personal belongings. Offer the family the opportunity to view the body.
**G**	**Give**	Give them your card and access information. Offer to answer any questions that may arise later. Always return their call.

**Table:** 

**Critical Actions:** Initiate appropriate management for septic shock and hypoxia. Recognize clinical need for intubation/mechanical ventilation. Phone power of attorney for patient unable to advocate for own wishes. Deploy communication framework, such as GRIEV_ING, to discuss goals of care. Provide appropriate de-escalation of care based on discussion with power of attorney.	0:00

**Table:** 

	Milestone	Did not achieve level 1	Level 1	Level 2	Level 3
1	**Emergency Stabilization (PC1)**	□ Did not achieve Level 1	□ Recognizes abnormal vital signs	□ Recognizes an unstable patient, requiring intervention Performs primary assessment Discerns data to formulate a diagnostic impression/plan	□ Manages and prioritizes critical actions in a critically ill patient Reassesses after implementing a stabilizing intervention
2	**Performance of focused history and physical (PC2)**	□ Did not achieve Level 1	□ Performs a reliable, comprehensive history and physical exam	□ Performs and communicates a focused history and physical exam based on chief complaint and urgent issues	□ Prioritizes essential components of history and physical exam given dynamic circumstances
3	**Diagnostic studies (PC3)**	□ Did not achieve Level 1	□ Determines the necessity of diagnostic studies	□ Orders appropriate diagnostic studies. Performs appropriate bedside diagnostic studies/procedures	□ Prioritizes essential testing Interprets results of diagnostic studies Reviews risks, benefits, contraindications, and alternatives to a diagnostic study or procedure
4	**Diagnosis (PC4)**	□ Did not achieve Level 1	□ Considers a list of potential diagnoses	□ Considers an appropriate list of potential diagnosis May or may not make correct diagnosis	□ Makes the appropriate diagnosis Considers other potential diagnoses, avoiding premature closure
5	**Pharmacotherapy (PC5)**	□ Did not achieve Level 1	□ Asks patient for drug allergies	□ Selects an medication for therapeutic intervention, consider potential adverse effects	□ Selects the most appropriate medication and understands mechanism of action, effect, and potential side effects Considers and recognizes drug-drug interactions
6	**Observation and reassessment (PC6)**	□ Did not achieve Level 1	□ Reevaluates patient at least one time during case	□ Reevaluates patient after most therapeutic interventions	□ onsistently evaluates the effectiveness of therapies at appropriate intervals
7	**Disposition (PC7)**	□ Did not achieve Level 1	□ Appropriately selects whether to admit or discharge the patient	□ Appropriately selects whether to admit or discharge Involves the expertise of some of the appropriate specialists	□ Educates the patient appropriately about their disposition Assigns patient to an appropriate level of care (ICU/Tele/Floor) Involves expertise of all appropriate specialists
9	**General Approach to Procedures (PC9)**	□ Did not achieve Level 1	□ Identifies pertinent anatomy and physiology for a procedure Uses appropriate Universal Precautions	□ Obtains informed consent Knows indications, contraindications, anatomic landmarks, equipment, anesthetic and procedural technique, and potential complications for common ED procedures	□ Determines a back-up strategy if initial attempts are unsuccessful Correctly interprets results of diagnostic procedure
20	**Professional Values (PROF1)**	□ Did not achieve Level 1	□ Demonstrates caring, honest behavior	□ Exhibits compassion, respect, sensitivity and responsiveness	□ Develops alternative care plans when patients’ personal beliefs and decisions preclude standard care
22	**Patient centered communication (ICS1)**	□ Did not achieve level 1	□ Establishes rapport and demonstrates empathy to patient (and family) Listens effectively	□ Elicits patient’s reason for seeking health care	□ Manages patient expectations in a manner that minimizes potential for stress, conflict, and misunderstanding. Effectively communicates with vulnerable populations, (at risk patients and families)
23	**Team management (ICS2)**	□ Did not achieve level 1	□ Recognizes other members of the patient care team during case (nurse, techs)	□ Communicates pertinent information to other healthcare colleagues	□ Communicates a clear, succinct, and appropriate handoff with specialists and other colleagues Communicates effectively with ancillary staff

**Table:** 

#1	Transthoracic echocardiogram showing cardiac standstill

**Table:** 

**Age**: 35	**Sex**: Male	**Method of Transportation**: EMS

**Figure:**
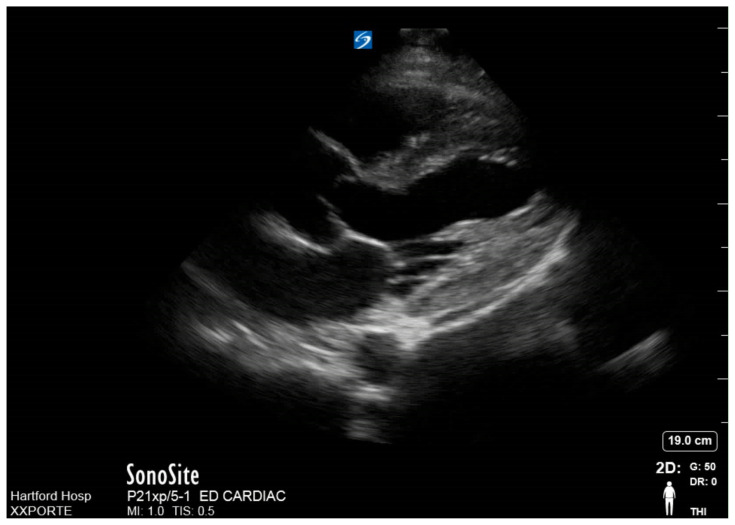


**Figure:**
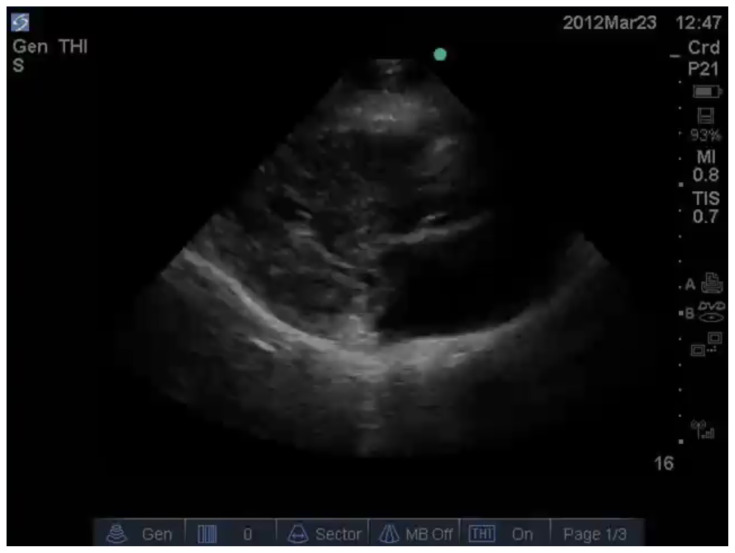


**Table:** 

*Learner Actions*	*Suggested Statements*
Learner meets with you.	Introduce yourself as the patient’s parent or sibling (depending on your age and your preference).
Learner describes the patient’s condition and ongoing resuscitation efforts.	Ask clarifying questions, especially if the learner uses too much technical terminology. Possible questions could include: - “What do you mean, cardiac arrest?” - “I don’t know what epinephrine is, doctor.”
Learners communicate the patient’s grim prognosis. They should clearly and unambiguously state that the patient will die.	Ask clarifying questions, especially if the learner is vague or imprecise. Possible questions could include: - “Can you get his heart restarted, doctor?” - “It sounds like you think he’s going to die, doctor.”
Learner invites you to witness the resuscitation and prepares you for the resuscitation environment.	You have a strong preference for witnessing the resuscitation. - “Yes, doctor. I’d like to see what’s going on.” - “So you’re saying that he will have a bunch of tubes and IVs connected to him, and someone will be pushing on his chest. I understand.”
Learner provides support while you are in the resuscitative environment.	You may wish to ask clarifying questions about the environment. If the learner has done a good job preparing you for the resuscitation environment, you will be prepared to terminate efforts. Provide positive reinforcement to the learners. - “What’s this tube sticking out of his mouth?” - “Yes, doctor, I’ve seen enough. I know that he’s gone. We can let him go now.” - “I appreciate you letting me be with him during his last moments.”

**Table:** 

*Learner Error*	*Suggested Statements*
Learner does not think to contact you until after resuscitation efforts are terminated.	You will be very upset to find that the patient has passed away. If learners display appropriate communication skills during death notification, you will remain calm and understanding. However, you should express regret that you “weren’t there for him at the end.” - “What do you mean, he’s dead? The ambulance just brought him here.” - “Did he wake up? Did he ask for me?” - “I wish I had a chance to say goodbye to him. He seemed to be doing better. This is so sudden.”
Learner meets with you while resuscitation is ongoing but does not offer to let you into the resuscitation bay.	You should ask to see the patient, for example by saying: “Can I go see him one last time?” - If learners allow you into the resuscitation bay, you can proceed with ideal scripting above. - If learners decline to let you witness the resuscitation, you will remain calm and understanding. However, you should express regret that you “weren’t there for him at the end.”
Learner does not exhibit an appropriate degree of empathy.	Attempt to slow the learner down and give them an opportunity to self-correct. Communicate your feelings to the learner, especially if they are offensive: - “I’m sorry, doctor. This is a lot to process. Can I have a moment?” - “Look, I know he’s just another drug addict to you, but he’s my family.”
Learner uses too much jargon or technical terminology.	- Ask clarifying questions about confusing terminology.
Learner is vague or imprecise.	Encourage clear and unambiguous language: - “You think that he’ll recover from this, right, doctor?” - “So if you can’t get his heart restarted, what will happen to him? Will he die?”
Learner does not prepare you adequately for the resuscitation environment.	Demonstrate to the learner that you are overwhelmed: - “I’m sorry, doctor. This is too much for me. I had better step out.” - “I wasn’t expecting all this commotion.” - “What are you doing to his chest? Isn’t that hurting him?”

**Table:** SIMULATION EVENTS TABLE

Minute (State)	Participant Action/Trigger	Patient Status (Simulator Response) & Operator Prompts	Monitor Display (Vital Signs)
0:00 (Base-line)	The learners take EMS report and begin patient assessment.	If learners attempt to communicate with the patient, the patient will be unresponsive.	PEA or asystole
0:10	Learners continue appropriate cardiac arrest care.	Learners should continue CPR, consider administration of ACLS medications such as epinephrine, and assess the patient’s access. The LMA is appropriately sized and providing effective ventilation. Learners may consider reversible etiologies of cardiac arrest and implement interventions accordingly, without change in patient status. Bedside cardiac ultrasound, if requested, will show cardiac standstill. End-tidal CO2, if requested, will be low.	PEA or asystole
5:00	Learners recognize high likelihood of poor patient prognosis.	Social work should notify learners that the patient’s family has arrived in the ED and is awaiting news. The standardized patient or case confederate playing the family member should be placed outside of the simulation lab to wait for learners.	Asystole
5:30	Learners form strategy for breaking bad news to family	At this stage of the case, learners may choose to manage the resuscitation and initiate family discussion in several ways:	Asystole
		(1) Learners may choose to end the resuscitation, then notify family of patient’s death. (2) Learners may choose to temporarily continue the resuscitation and initiate family discussion in a separate space. However, they may decline the family’s request to “see the patient” prior to terminating resuscitation efforts. (3) Learners may choose to employ familywitnessed resuscitation.	
(Case Completion)	Family discussion completed	Effective communication will allow family to accept the patient’s death. However, depending on learner choices, the family member will either express regret that they “weren’t there for [the patient],” or express gratitude that “you took such good care of [the patient].”	Asystole

**Table 2:** The GRIEV_ING Mnemonic Source: Hobgood *et al*, 2004

**G**	**Gather**	Gather the family; ensure that all members are present.
**R**	**Resources**	Call for support resources available to assist the family with their grief, ie, chaplain services, ministers, family, and friends.
**I**	**Identify**	Identify yourself, identify the deceased or injured patient by *name*, and identify the state of knowledge of the family relative to the events of the day.
**E**	**Educate**	Briefly educate the family as to the events that have occurred in the emergency department; educate them about the state of their loved one.
**V**	**Verify**	Verify that their family member has died. *Be clear!* Use the words “dead” or “died.”
**__**	**Space**	Give the family personal space and time for an emotional moment; allow the family time to absorb the information.
**I**	**Inquire**	Ask if there are any questions and answer them all.
**N**	**Nuts and Bolts**	Inquire about organ donation, funeral services, and personal belongings. Offer the family the opportunity to view the body.
**G**	**Give**	Give them your card and access information. Offer to answer any questions that may arise later. Always return their call.

**Table:** 

**Critical Actions:** Provide appropriate cardiac arrest management for a patient in PEA/asystole. Recognize indicators of poor prognosis in cardiac arrest, such as cardiac standstill on bedside ultrasound. Take steps to notify family of expected death. Deploy communication framework, such as GRIEV_ING, to discuss poor prognosis with family. Invite family to witness resuscitation prior to termination of efforts.	0:00

**Table:** 

	Milestone	Did not achieve level 1	Level 1	Level 2	Level 3
1	**Emergency Stabilization (PC1)**	□ Did not achieve Level 1	□ Recognizes abnormal vital signs	□ Recognizes an unstable patient, requiring intervention Performs primary assessment Discerns data to formulate a diagnostic impression/plan	□ Manages and prioritizes critical actions in a critically ill patient Reassesses after implementing a stabilizing intervention
2	**Performance of focused history and physical (PC2)**	□ Did not achieve Level 1	□ Performs a reliable, comprehensive history and physical exam	□ Performs and communicates a focused history and physical exam based on chief complaint and urgent issues	□ Prioritizes essential components of history and physical exam given dynamic circumstances
3	**Diagnostic studies (PC3)**	□ Did not achieve Level 1	□ Determines the necessity of diagnostic studies	□ Orders appropriate diagnostic studies. Performs appropriate bedside diagnostic studies/procedures	□ Prioritizes essential testing Interprets results of diagnostic studies Reviews risks, benefits, contraindications, and alternatives to a diagnostic study or procedure
4	**Diagnosis (PC4)**	□ Did not achieve Level 1	□ Considers a list of potential diagnoses	□ Considers an appropriate list of potential diagnosis May or may not make correct diagnosis	□ Makes the appropriate diagnosis Considers other potential diagnoses, avoiding premature closure
5	**Pharmacotherapy (PC5)**	□ Did not achieve Level 1	□ Asks patient for drug allergies	□ Selects an medication for therapeutic intervention, consider potential adverse effects	□ Selects the most appropriate medication and understands mechanism of action, effect, and potential side effects Considers and recognizes drug-drug interactions
6	**Observation and reassessment (PC6)**	□ Did not achieve Level 1	□ Reevaluates patient at least one time during case	□ Reevaluates patient after most therapeutic interventions	□ onsistently evaluates the effectiveness of therapies at appropriate intervals
7	**Disposition (PC7)**	□ Did not achieve Level 1	□ Appropriately selects whether to admit or discharge the patient	□ Appropriately selects whether to admit or discharge Involves the expertise of some of the appropriate specialists	□ Educates the patient appropriately about their disposition Assigns patient to an appropriate level of care (ICU/Tele/Floor) Involves expertise of all appropriate specialists
9	**General Approach to Procedures (PC9)**	□ Did not achieve Level 1	□ Identifies pertinent anatomy and physiology for a procedure Uses appropriate Universal Precautions	□ Obtains informed consent Knows indications, contraindications, anatomic landmarks, equipment, anesthetic and procedural technique, and potential complications for common ED procedures	□ Determines a back-up strategy if initial attempts are unsuccessful Correctly interprets results of diagnostic procedure
20	**Professional Values (PROF1)**	□ Did not achieve Level 1	□ Demonstrates caring, honest behavior	□ Exhibits compassion, respect, sensitivity and responsiveness	□ Develops alternative care plans when patients’ personal beliefs and decisions preclude standard care
22	**Patient centered communication (ICS1)**	□ Did not achieve level 1	□ Establishes rapport and demonstrates empathy to patient (and family) Listens effectively	□ Elicits patient’s reason for seeking health care	□ Manages patient expectations in a manner that minimizes potential for stress, conflict, and misunderstanding. Effectively communicates with vulnerable populations, (at risk patients and families)
23	**Team management (ICS2)**	□ Did not achieve level 1	□ Recognizes other members of the patient care team during case (nurse, techs)	□ Communicates pertinent information to other healthcare colleagues	□ Communicates a clear, succinct, and appropriate handoff with specialists and other colleagues Communicates effectively with ancillary staff

## INSTRUCTOR MATERIALS

**Case 1 Title:** Connecting Remotely

**Case Description & Diagnosis (short synopsis):** This case features an 80-year-old female with multiple comorbidities who presents to the emergency department in septic shock due to multifocal pneumonia. Learners must recognize the patient’s unsustainable work of breathing, which would necessitate intubation to ensure patient survival. However, the patient’s wishes are to avoid intubation or other invasive procedures, which participants will discover if they contact the patient’s power of attorney (POA). Learners should call the patient’s POA to discuss code status and goals of care, thereby honoring the patient’s wishes about her end-of-life care.

**Equipment or Props Needed:**

High-fidelity simulator/mannequin
Vital sign monitor
Monitor to show imaging and lab results
Intubation equipment with direct laryngoscopy and/or video laryngoscopy
Supplemental oxygen devices

**Confederates needed:**

One standardized patient or case confederate to play the patient’s POA, who can be a son, daughter, sibling, or spouse depending on the individual’s age and gender. Since communication occurs over the phone in this case, this role can also be played by the instructor if desired.

**Stimulus Inventory:**

**Background and brief information:** The 80-year-old patient is brought to the ED by emergency medical services (EMS) from her assisted living facility. This morning, the patient did not attend community activities, which was unusual for her. Staff members subsequently found the patient altered on the ground of her living unit. She was noted to be tachycardic and hypotensive by EMS.

**Initial presentation:** On arrival, the patient appears altered and is unable to answer questions, though she moans and spontaneously moves all extremities. She is febrile, tachycardic, hypoxic, and hypotensive.

**How the scene unfolds:** The patient’s unstable vital signs will improve with non-invasive measures, such as fluid resuscitation and supplemental oxygenation, though her work of breathing will not normalize. Chest X-ray will demonstrate multifocal pneumonia. Learners should recognize life-threatening septic shock. The patient’s survival will be dependent on mechanical ventilation and prolonged intensive care unit (ICU) admission.

*If learners recognize the need for early goals of care discussion*: Learners will be provided with the phone number for the patient’s POA, who is to be played by the standardized patient or case confederate. The POA will state that the patient is highly independent and values her quality of life. She had a prior hospitalization 2 months ago for a fractured hip that necessitated operative repair and a prolonged stay in inpatient rehabilitation. The patient has previously stated to her POA that she would not want to go through that again and is in the process of filing “do not resuscitate” paperwork. Learners should de-escalate care according to the patient’s established wishes.

*If participants pursue invasive procedures, such as intubation, without initiating a goals of care discussion*: After the procedure, the patient’s POA will call, highly upset that the patient was intubated. The POA will state that the patient “never wanted to be on a ventilator,” “valued her independence,” and “always told me to let her go if she was going to be too sick to live by herself at home.”

**Critical actions:**

1. Initiate appropriate management for septic shock and hypoxia
2. Recognize clinical need for intubation / mechanical ventilation
3. Phone power of attorney for patient unable to advocate for own wishes
4. Deploy communication framework, such as GRIEV_ING, to discuss goals of care
5. Provide appropriate de-escalation of care based on discussion with power of attorney

**Case 1 Title:** Connecting Remotely

**Chief Complaint:** Altered mental status.

**General Appearance:** ill-appearing, confused, moving extremities but not to command

**Primary Survey:**

- **Airway:** Intact
- **Breathing:** Intact
- **Circulation:** Intact, 2+ pulses throughout

**History:**

- **History of present illness:** This history is provided by EMS because the patient is too altered to participate in the interview. The patient is an 80-year-old female presenting to the ED with altered mental status from her assisted living facility. The patient had recently returned to her facility after a prolonged stay in rehabilitation after a hip fracture 2 months ago. She did not come to community activities that morning, which was unusual for her. Staff members discovered the patient on the floor of her apartment. At baseline, she is alert and oriented x4, but currently, she is only making confused sounds. Noted to be tachycardic and hypotensive by EMS.
- **Past medical history:** Hypertension, hyperlipidemia, coronary artery disease, myocardial infarction with remote stent placement, congestive heart failure, chronic kidney disease
- **Past surgical history:** Appendectomy (remote)
- **Medications:** Aspirin, lisinopril, carvedilol, simvastatin, furosemide
- **Allergies:** none
- **Social history:** No known alcohol, tobacco, or illicit use
- **Family history:** Non-contributory

**Secondary Survey/Physical Examination:**

- **General appearance:** Ill-appearing, confused, verbalizing in response to questions, but unable to form coherent speech.
- **HEENT:**
  ○ **Head:** Within normal limits (wnl)
  ○ **Eyes:** Eyelids half-closed on manikin, pupils equally round and reactive.
  ○ **Ears:** wnl
  ○ **Nose:** wnl
  ○ **Throat:** wnl
- **Neck:** wnl
- **Heart:** Regular rate and rhythm. Tachycardic.
- **Lungs:** Bilateral crackles. Tachypneic.
- **Abdominal/GI:** wnl
- **Genitourinary:** wnl
- **Rectal:** wnl
- **Extremities:** wnl
- **Back:** wnl
- **Neuro:** Cranial nerves grossly intact. Moving all extremities restlessly and spontaneously, but not to command.
- **Skin:** Cyanotic; no rashes, ulcerations, or wounds.
- **Lymph:** wnl
- **Psych:** Unable to assess.

*Electrocardiogram*: sinus tachycardia

Ewingdo. ECG sinus tachycardia. In: Wikimedia Commons. https://commons.wikimedia.org/wiki/File:ECG_Sinus_Tachycardia_132_bpm.jpg Published November 13, 2020. Accessed August 24, 2021. CC BY-SA 4.0.

*Chest X-Ray*: multifocal pneumonia

Franquet T, Chung JH. X-ray of bronchopneumonia. In: Wikimedia Commons. https://commons.wikimedia.org/wiki/File:X-ray_of_bronchopneumonia.png#/media/File:Xray_of_bronchopneumonia.png Published February 20, 2019. Accessed August 24, 2021. CC BY-SA 4.0.

## STANDARDIZED PATIENT BRIEFING MATERIALS

### Case 1: Connecting Remotely

#### Case Background

You are the family member of a critically ill patient in the emergency department (ED) who is suffering from a life-threatening pneumonia. The patient is elderly, with a medical history of significant heart and kidney issues. She is unlikely to survive this illness without placement on a ventilator and intensive care unit (ICU) admission. Even if she survives, she is at very high risk for decreased independence, mobility, and quality of life.

#### Relationship to the Patient / Additional Context

- Depending on your age and preferences, you may be the patient’s significant other, sibling, or child.
- Two months ago, the patient had a fall in her assisted living facility and broke her hip. She had to undergo surgery for a hip replacement. Recovery from surgery was very difficult. She lost mobility and strength while she was hospitalized and regaining her independence required a prolonged stay in a rehabilitation center.
- The patient has expressed to you on multiple occasions that she “never wants to go through that again.” She values being able to live at home and take care of herself. She strongly disliked being in bed and relying on others to care for her basic needs.
- The patient has been in communication with her primary care physician to complete “do not resuscitate / do not intubate” paperwork. You are not sure if she has finished it yet.

#### Your Role in the Case

The learners may contact you by phone to discuss the patient’s illness, prognosis, and wishes for her life and death. You should do your best to accurately represent the patient’s wants and needs. She has been very clear with you that she found rehabilitation traumatizing, and she would not want life-prolonging measures if she would experience a decline in her quality of life.

#### Ideal Case Flow

The learner will provide you with a clear and unambiguous explanation of the patient’s current condition and prognosis, while displaying an appropriate degree of empathy. They will ask about the patient’s wishes for her life and death. Using the information you provide, they will suggest a treatment plan that you feel represents the patient’s wishes. Most likely, their suggested treatment plan will involve palliative care, comfort care measures, or de-escalation of care.

### Some suggested conversation scripting is below

#### Learner Pitfalls

The learner may do an ineffective job of communicating with you. Some possible errors and conversational scripting are outlined below:

## OPERATOR MATERIALS

**Diagnosis:**

Septic shock

**Disposition:**

ICU or appropriate palliative setting

## DEBRIEFING AND EVALUATION PEARLS

### Case 1: Connecting Remotely

**Pearls:** Older patients with serious comorbidities, such as heart failure and chronic kidney disease, have high ED utilization rates in the last 6 months of life. This patient population is vulnerable to decreased activities of daily living and loss of functional status due to disability from chronic illness. Additionally, they experience high mortality rates after intubation and CPR. Despite being at high risk for decreased quality of life with each episode of illness, most older adults do not have advance directives available at ED presentation.

Goals of care discussions during a severe, life-threatening episode of illness are particularly challenging due to a variety of factors, including limited time to establish the patient-physician relationship and form shared consensus about crucial decisions. In the ED setting, physicians are often forced to evaluate critically ill patients for time-sensitive interventions, such as intubation and mechanical ventilation, without guidance from advance directives. To navigate these tricky situations, EM physicians should prioritize understanding a patient’s wishes in life and death to ensure alignment of treatments and interventions with patient values.

#### Other debriefing points

As part of debriefing, facilitators should provide feedback on the effectiveness of the learner’s communication with the standardized patient or case confederate. Discussion points may include:

- Use of a communication strategy, such as GRIEV_ING or SPIKES. (We used GRIEV_ING to frame the debriefing, but other approaches would be reasonable.)
- Setting the stage for an empathetic and effective high-stakes discussion over the phone.
- Use of clear and unambiguous language when describing the patient’s clinical condition.
- Providing empathetic responses to family concerns.
- Allowing time for emotional processing from family.
- Forming an accurate view of the patient’s end-of-life wishes, or her values about what she would consider an acceptable quality of life.
- Use of clear and unambiguous language when agreeing on next steps with family, such as comfort measures or de-escalation of care.

In our experience running the case, most learner groups elected to intubate the patient prior to initiating goals of care discussion with the patient’s POA; when asked to describe their decision-making, most learner groups cited the patient’s critical illness and the need for rapid life-sustaining intervention. This led to interesting debriefing discussions about the EM physician’s role in ensuring patient-centered outcomes. Facilitators found the following inquiries effective in eliciting open-ended discussion between learners:

- “Have you encountered a critically ill older patient in septic shock before? Tell me about what happened to that patient during their hospital course.”
- “I’d like you to think about the patient in this simulation case, who was an elderly patient in septic shock. What do you think this patient’s likely outcome is, assuming she receives maximal medical intervention?”
- “Do you think that this outcome is in alignment with the patient’s values regarding her life and death?”
- “What are the consequences of delaying a time-sensitive but invasive intervention? What are the consequences of committing a patient to a treatment pathway that does not align with their end-of-life values? How do we balance between these concerns?”
- “When we are unsure of a patient’s values, are there any sources that we can look to?”
- “What barriers do we encounter when we attempt to establish a patient’s values regarding their life and death?”
- “What resources do we have in our ED setting for managing these barriers?”

#### Wrap Up

We offered the following resources to learners for further reading: Hobgood C, Harward D, Newton K, Davis W. The educational intervention “GRIEV_ING” improves the death notification skills of residents. *Acad Emerg Med*. 2004;12(4):296–301. doi:10.1197/j.aem.2004.12.008

Ouchi K, George N, Schuur JD, et al. Goals-of-care conversations for older adults with serious illness in the emergency department: challenges and opportunities. *Ann Emerg Med*. 2019;74(2):276–84. doi:10.1016/j.annemergmed.2019.01.003

## SIMULATION ASSESSMENT

*Case 1: Connecting Remotely*

Learner: _________________________________________

***Assessment Timeline***

This timeline is to help observers assess their learners. It allows observer to make notes on when learners performed various tasks, which can help guide debriefing discussion.

**Critical Actions:**

□ Initiate appropriate management for septic shock and hypoxia.
□ Recognize clinical need for intubation/ mechanical ventilation.
□ Phone power of attorney for patient unable to advocate for own wishes.
□ Deploy communication framework, such as GRIEV_ING, to discuss goals of care.
□ Provide appropriate de-escalation of care based on discussion with power of attorney.

**Summative and formative comments:**

**Milestones assessment:**

## INSTRUCTOR MATERIALS

**Case 2 Title:** Family Witnessed Resuscitation

**Case Description & Diagnosis (short synopsis):** This case features a 35-year-old male with a history of opioid use disorder presenting to the Emergency Department in cardiac arrest with unknown downtime. Throughout his resuscitation, he is in pulseless electrical activity (PEA) or asystole. Learners must recognize the futility of further resuscitation and take steps to prepare the family for the patient’s death. Ideally, participants will invite family to witness the resuscitation prior to termination of efforts.

**Equipment or Props Needed:**

High fidelity simulator/mannequin
Vital sign monitor
Monitor to show imaging and lab results
Intubation equipment with direct laryngoscopy and/or video laryngoscopy
Defibrillator
Supplemental oxygen devices

**Confederates needed:**

One standardized patient or case confederate to play the patient’s family member, who can be a parent or sibling depending on the individual’s age and gender.

**Stimulus Inventory:**

**Background and brief information:** The 35-year-old patient is brought to the ED by EMS from his family’s home. EMS will report that the patient was found in cardiac arrest by family members. Initial rhythm was noted to be pulseless electrical activity (PEA).

**Initial presentation:** Patient is unresponsive with cold and mottled extremities. His pupils are fixed and dilated. He has an appropriately sized LMA (laryngeal mask airway) in place.

**How the scene unfolds:** Despite all resuscitation efforts, the patient will remain in PEA or asystole throughout the case, with cardiac standstill on bedside echocardiogram. The ED social worker will inform learners that the patient’s family member has arrived in the ED and is awaiting news. Learners should recognize medical futility of further resuscitation efforts and take steps to notify the family member of the patient’s poor prognosis.

*If learners choose to terminate resuscitation efforts, then deliver death notification to the family:*

Appropriate communication and deployment of a structured approach to breaking bad news, such as GRIEV_ING, will allow the family member to accept the news calmly. However, the family will express regret that he/she was not there for the patient’s final moments.

*If learners choose to temporarily continue resuscitation efforts and discuss the patient’s prognosis with the family in a separate space:* Appropriate communication and deployment of a structured approach to breaking bad news, such as GRIEV_ING, will prepare the family member for the patient’s imminent death. The family member will ask to see the patient.

*If learners choose to employ family witnessed resuscitation*: Appropriate communication and deployment of a structured approach to breaking bad news, such as GRIEV_ING, will prepare the family member to calmly observe resuscitation efforts and accept the patient’s imminent death. After the patient is pronounced, the family member will express gratitude that they were there for the patient’s final moments.

**Critical actions:**

1. Provide appropriate cardiac arrest management for a patient in PEA/asystole
2. Recognize indicators of poor prognosis in cardiac arrest, such as cardiac standstill on bedside ultrasound
3. Take steps to notify family of expected death
4. Deploy communication framework, such as GRIEV_ING, to discuss poor prognosis with family
5. Invite family to witness resuscitation prior to termination of efforts

**Case 2 Title:** Family Witnessed Resuscitation

**Chief Complaint:** Cardiac arrest

**Vitals:** Asystole

**General Appearance:** Unresponsive, pale, cyanotic, closed eyes, fixed and dilated pupils

**Primary Survey:**

**Airway:** Appropriately sized LMA in place
**Breathing:** No spontaneous respirations
**Circulation:** No palpable peripheral or central pulses

**History:**

**History of present illness:** 35-year-old male with history of opioid use disorder presents to the ED in cardiac arrest. He was found at home by family members with unknown downtime, last seen normal approximately 2 hours prior to being found. Bystander cardiopulmonary resuscitation (CPR) was initiated. EMS states that the patient had a prior history of heroin use but had “gotten clean” recently. Initial rhythm was PEA. The patient has received a total of 20 minutes of CPR, including bystander CPR. Additionally, EMS administered 2 rounds of epinephrine and placed a laryngeal mask airway (LMA) on scene.
**Past medical history:** Opioid use disorder
**Past surgical history:** none
**Medications:** none
**Allergies:** none
**Social history:** Prior history of opioid use, largely prescription narcotics and heroin. Occasional tobacco use. Occasional alcohol use.
**Family history:** Non-contributory

**Secondary Survey/Physical Examination:**

**General appearance:** Unresponsive, pale, cyanotic.
**HEENT:**
  ○ **Head:** wnl
  ○ **Eyes:** Eyelids closed, fixed and dilated pupils.
  ○ **Ears:** wnl
  ○ **Nose:** wnl
  ○ **Throat:** Appropriately sized LMA in place.
**Neck:** wnl
**Heart:** No spontaneous cardiac activity.
**Lungs:** No spontaneous respirations.
**Abdominal/GI:** wnl
**Genitourinary:** wnl
**Rectal:** wnl
**Extremities:** wnl
**Back:** wnl
**Neuro:** No spontaneous motor activity, unresponsive to verbal or painful stimuli.
**Skin:** Cold, mottled, cyanotic.
**Lymph:** wnl
**Psych:** Unable to assess.

*Transthoracic echocardiogram / parasternal long:* cardiac standstill (indicator right)

Author’s own image

## STANDARDIZED PATIENT BRIEFING MATERIALS

### Case 2: Family Witnessed Resuscitation

#### Case Background

You are the family member of a patient who was brought to the ED in cardiac arrest. The patient has a longstanding history of prescription narcotic and heroin addiction, but recently had been in recovery. The cardiac arrest likely occurred due to a relapse in heroin use, leading to a fatal overdose. The patient exhibits no signs of life for the paramedics or in the ED, despite maximal resuscitation measures, and is expected to die from his overdose.

#### Relationship to the Patient / Additional Context

- Depending on your age and preferences, you may be the patient’s sibling or parent.
- The patient became addicted to prescription opioids after a wisdom tooth surgery 10 years ago. He started off buying Percocet and Norco off the street, then progressed to heroin abuse.
- In prior years, your relationship with the patient had been shaky. He sometimes stole from you and other family members, and he was sometimes homeless.
- The patient recently went to rehabilitation and seemed to be in recovery from his addiction. You had been allowing him to live in your home to get his feet under him. You hadn’t noticed any suspicious behavior that made you suspect he was using drugs again.
- You last spoke to the patient during dinner. You went to check on him, and found him in cardiac arrest, with no breathing and no pulse. You called 9-1-1 and they walked you through how to perform cardiopulmonary resuscitation (CPR).
- When the ambulance came, the paramedics told you which hospital the patient was going to. You drove immediately to the hospital to wait for news.

#### Your Role in the Case

The learners may meet with you to discuss the patient’s prognosis and likely death. Depending on how they choose to approach the conversation, they may meet with you after they have already terminated resuscitation efforts, or while resuscitation efforts are ongoing. You will accept the news calmly as long as the learners use effective and empathetic communication techniques.

#### Ideal Case Flow

The learner will meet with you while resuscitation efforts are still ongoing. They will provide you with a clear and unambiguous explanation of the patient’s grim prognosis, while displaying an appropriate degree of empathy. They will invite you to observe the resuscitation efforts and emotionally prepare you to witness cardiac arrest care.

**Some suggested conversation scripting is below:**

#### Learner Pitfalls

The learner may do an ineffective job of communicating with you. Some possible errors and conversational scripting are outlined below:

## OPERATOR MATERIALS

**Diagnosis:**

Cardiac Arrest

**Disposition:**

Expired

## DEBRIEFING AND EVALUATION PEARLS

### Case 2: Family Witnessed Resuscitation

**Pearls:** Family-witnessed resuscitation (FWR) describes the inclusion of family members during resuscitation efforts. In the ED, FWR may describe inviting the family to observe cardiac arrest care or other critical interventions, such as intubation. In recent years, FWR has gained broad support among many professional bodies, including the American Heart Association. Many studies demonstrate decreased bereavement-related depression and post-traumatic stress disorder (PTSD) symptoms among family members who were offered the choice of FWR. Additionally, FWR has not been shown to negatively impact resuscitation outcomes.

Effective deployment of FWR can be challenging, especially in the fast-paced and sometimes resource-limited environment of the ED. Considerations may include staff attitudes and beliefs surrounding the practice, staff knowledge and training, the presence of a dedicated staff member to support family, the selection of a suitable family member, and the physical space in which resuscitation is taking place. Physicians should utilize good judgment and effective communication skills to navigate these concerns when they offer FWR, to fully realize its potential benefits to family members.

#### Other debriefing points

As part of debriefing, facilitators should provide feedback on the effectiveness of the learner’s communication with the standardized patient or case confederate. Discussion points may include:

- Use of a communication strategy, such as GRIEV_ING or SPIKES. (We used GRIEV_ING to frame the debriefing, but other approaches would be reasonable.)
- Setting the stage for an empathetic and effective high-stakes discussion about the patient’s imminent death.
- Use of clear and unambiguous language when describing the patient’s clinical condition.
- Use of clear and unambiguous language when preparing family for witnessing events in the resuscitation bay.
- Providing empathetic responses to family concerns.
- Allowing time for emotional processing from family.
- Use of clear and unambiguous language when agreeing on next steps with family, such as termination of resuscitative efforts.

In our experience running the case, some learner groups were not aware of the practice of FWR; as a result, it did not occur to them to offer this option to the family. This triggered an opportunity for learner education and discussion of follow-up reading.

**Wrap Up:**

We offered the following resources to learners for further reading:

Farah MM, Thomas CA, Shaw KN. Evidence-based guidelines for family presence in the resuscitation room: a step-by-step approach. *Pediatr Emerg Care*. 2007;23(8):587–591.

Jabre P, Belpomme V, Azoulay E, et al. Family presence during cardiopulmonary resuscitation. *New Engl J Med*. 2013;368:1008–1018.

## SIMULATION ASSESSMENT

*Case 2: Family Witnessed Resuscitation*

Learner: _________________________________________

***Assessment Timeline***

This timeline is to help observers assess their learners. It allows observer to make notes on when learners performed various tasks, which can help guide debriefing discussion.

**Critical Actions:**

□ Provide appropriate cardiac arrest management for a patient in PEA/asystole.
□ Recognize indicators of poor prognosis in cardiac arrest, such as cardiac standstill on bedside ultrasound.
□ Take steps to notify family of expected death.
□ Deploy communication framework, such as GRIEV_ING, to discuss poor prognosis with family.
□ Invite family to witness resuscitation prior to termination of efforts.

**Summative and formative comments:**

**Milestones assessment:**
