# Supplementary material for: Breaking Bad News in the Emergency Department
Source: J Educ Teach Emerg Med. 2022 Apr 15;7(2):S1–S47. doi: 10.21980/J81W7H (PMC10332750; doi:10.21980/J81W7H)
Supplement: Supplementary file 1 [file JETem-7-2-S1-supp1.pptx]

## Slide 1
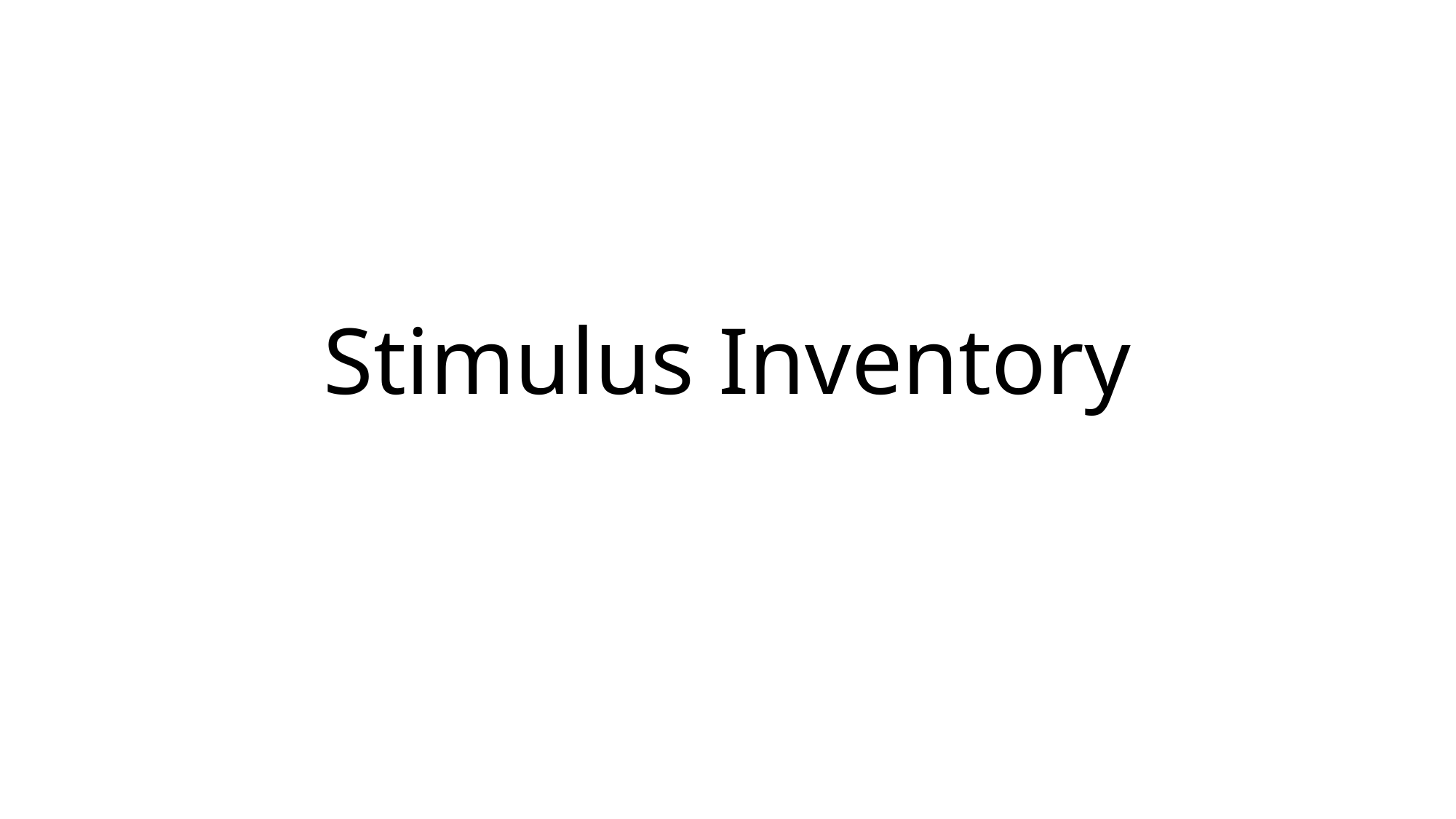

# Stimulus Inventory

## Slide 2
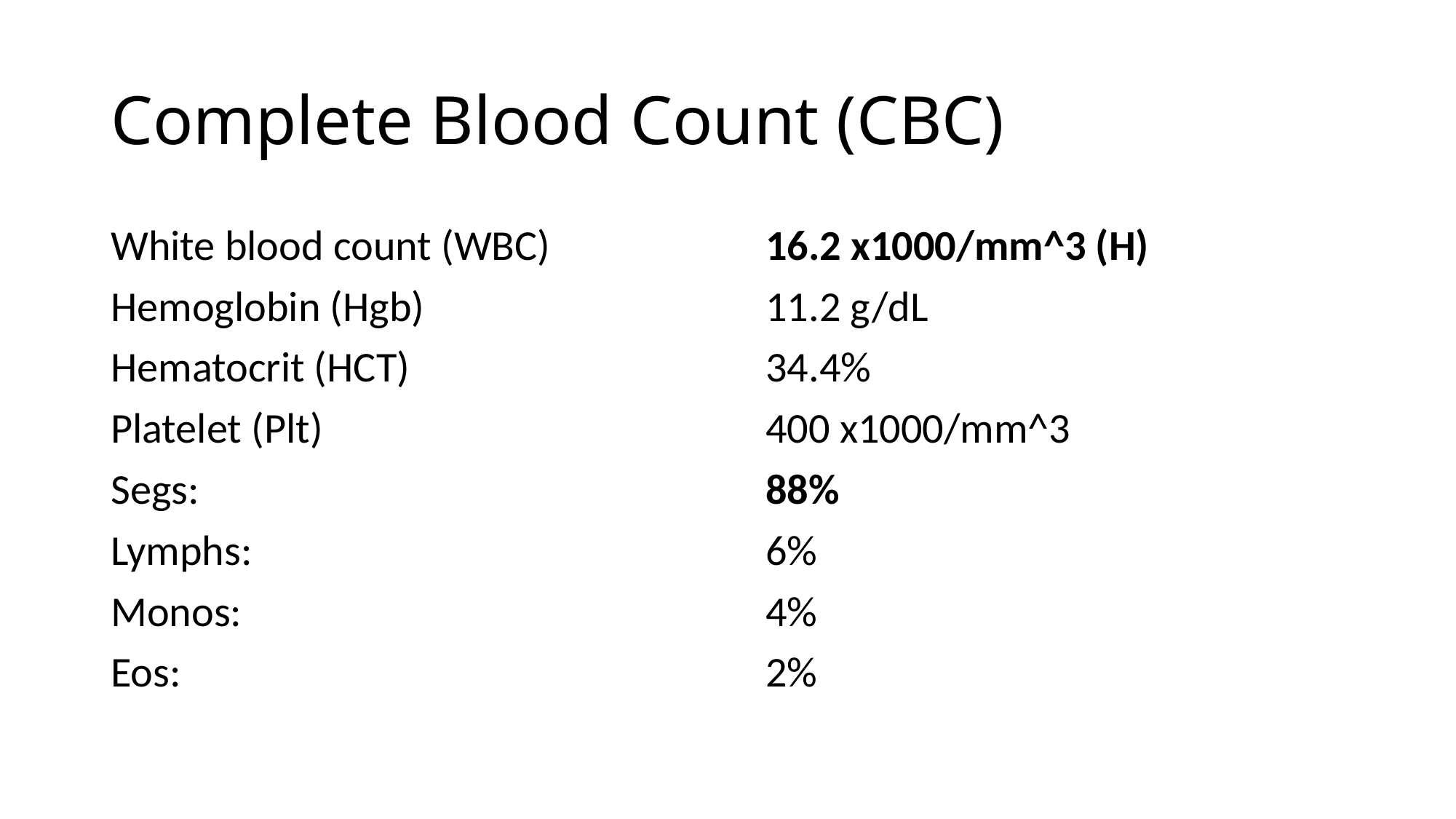

# Complete Blood Count (CBC)
White blood count (WBC) 		16.2 x1000/mm^3 (H)
Hemoglobin (Hgb)				11.2 g/dL
Hematocrit (HCT)				34.4%
Platelet (Plt)					400 x1000/mm^3
Segs:						88%
Lymphs:					6%
Monos:					4%
Eos:						2%

## Slide 3
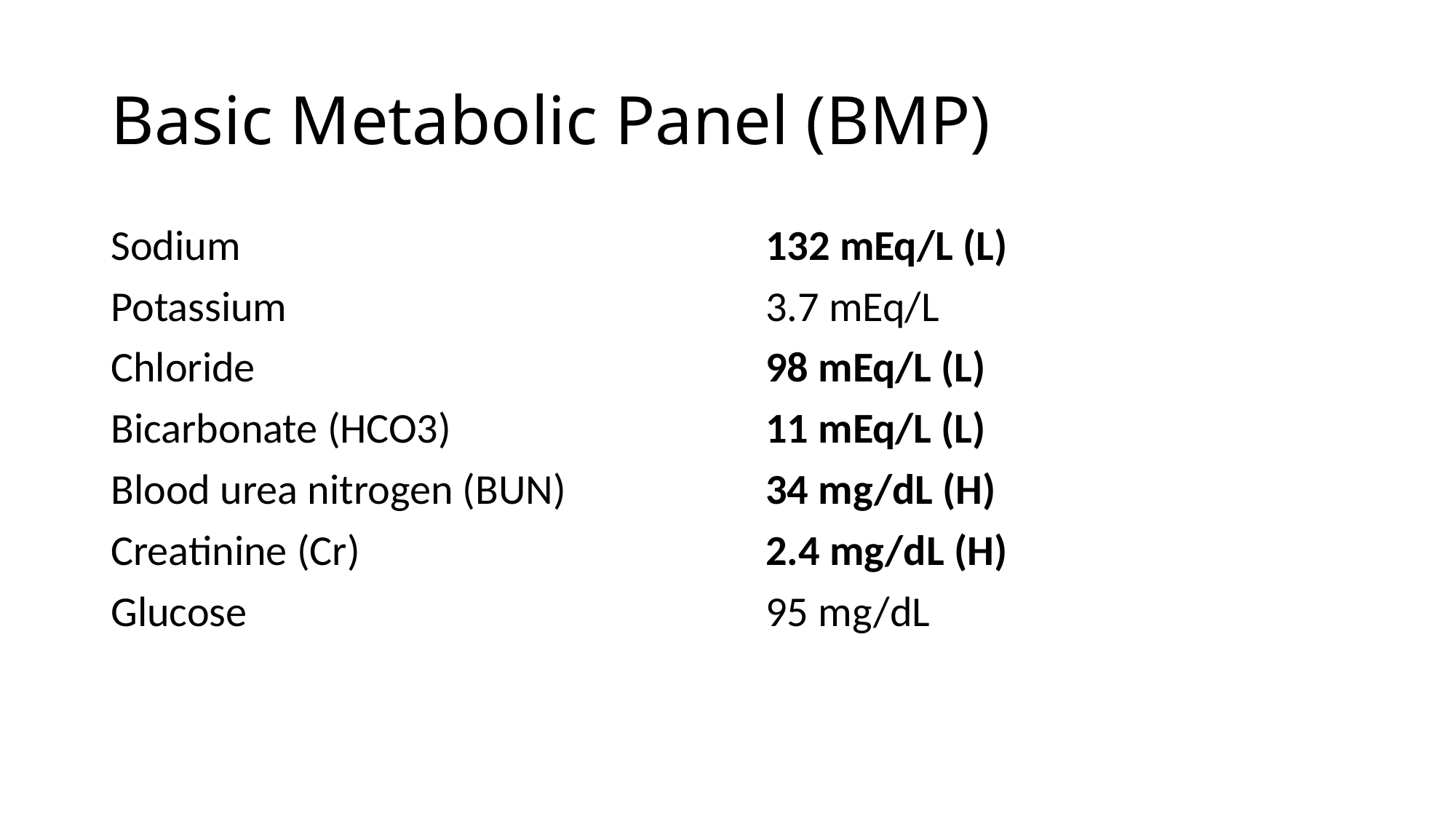

# Basic Metabolic Panel (BMP)
Sodium					132 mEq/L (L)
Potassium					3.7 mEq/L
Chloride					98 mEq/L (L)
Bicarbonate (HCO3)			11 mEq/L (L)
Blood urea nitrogen (BUN)		34 mg/dL (H)
Creatinine (Cr)				2.4 mg/dL (H)
Glucose 					95 mg/dL

## Slide 4
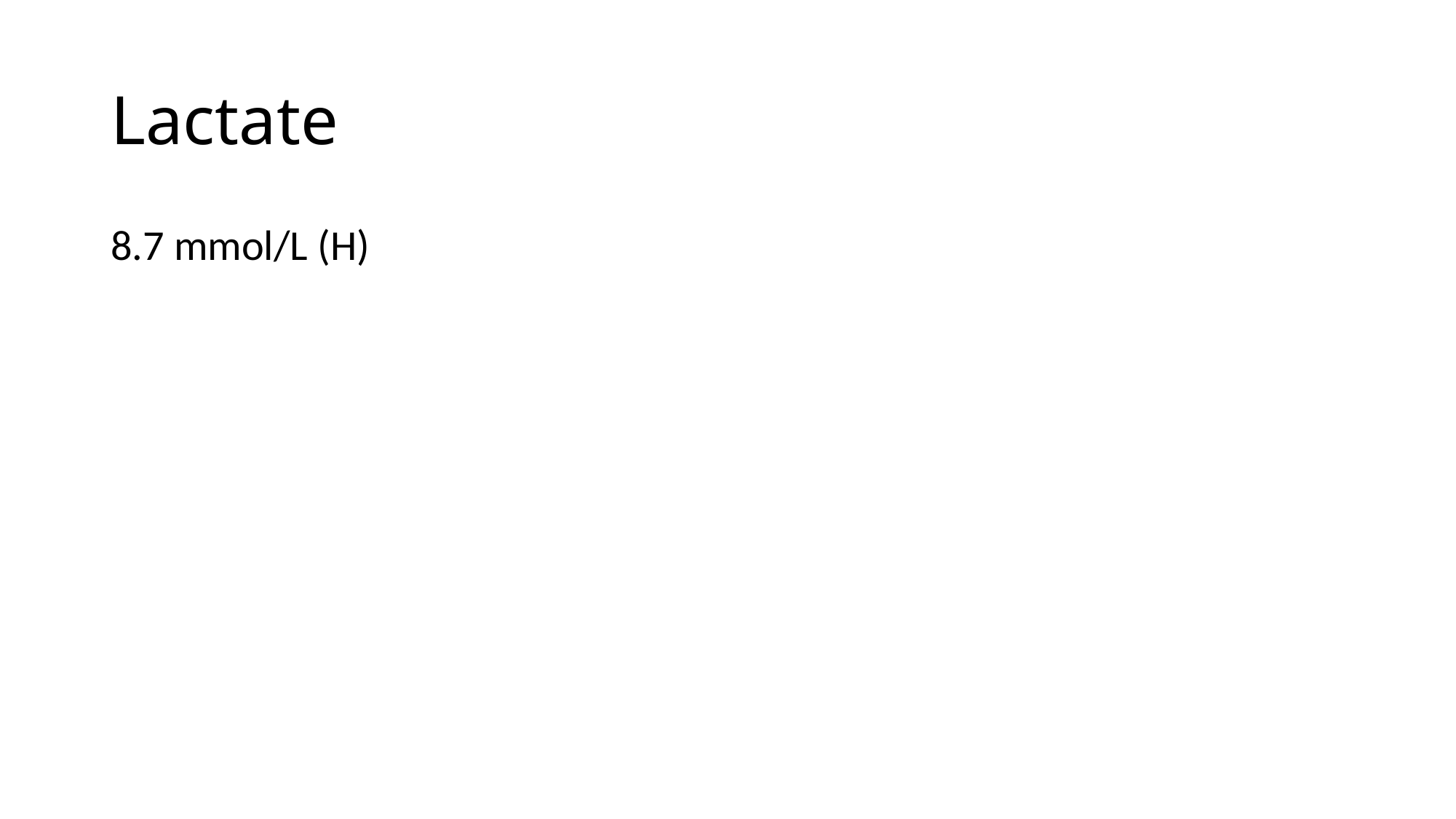

# Lactate
8.7 mmol/L (H)

## Slide 5
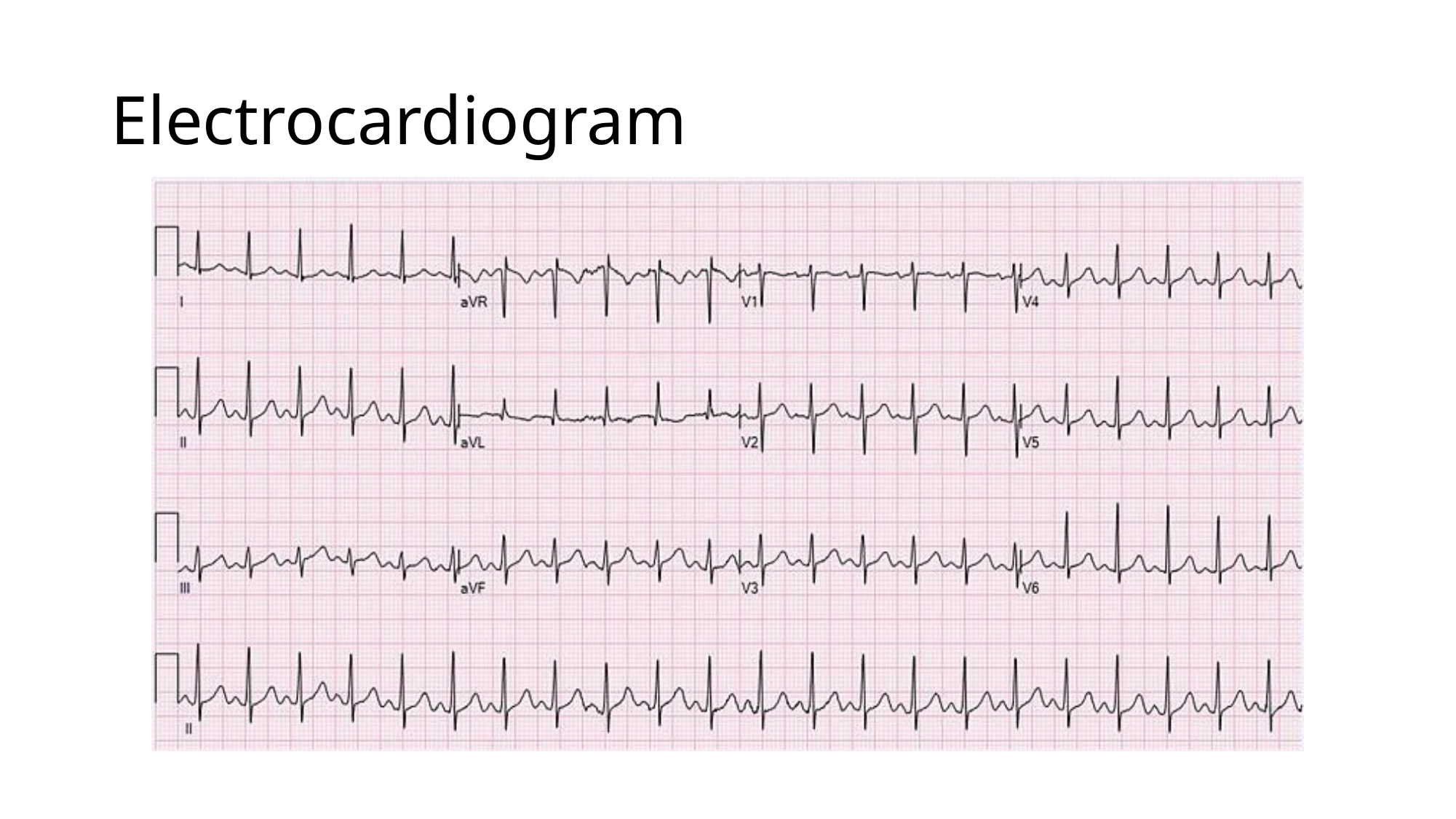

# Electrocardiogram

## Slide 6
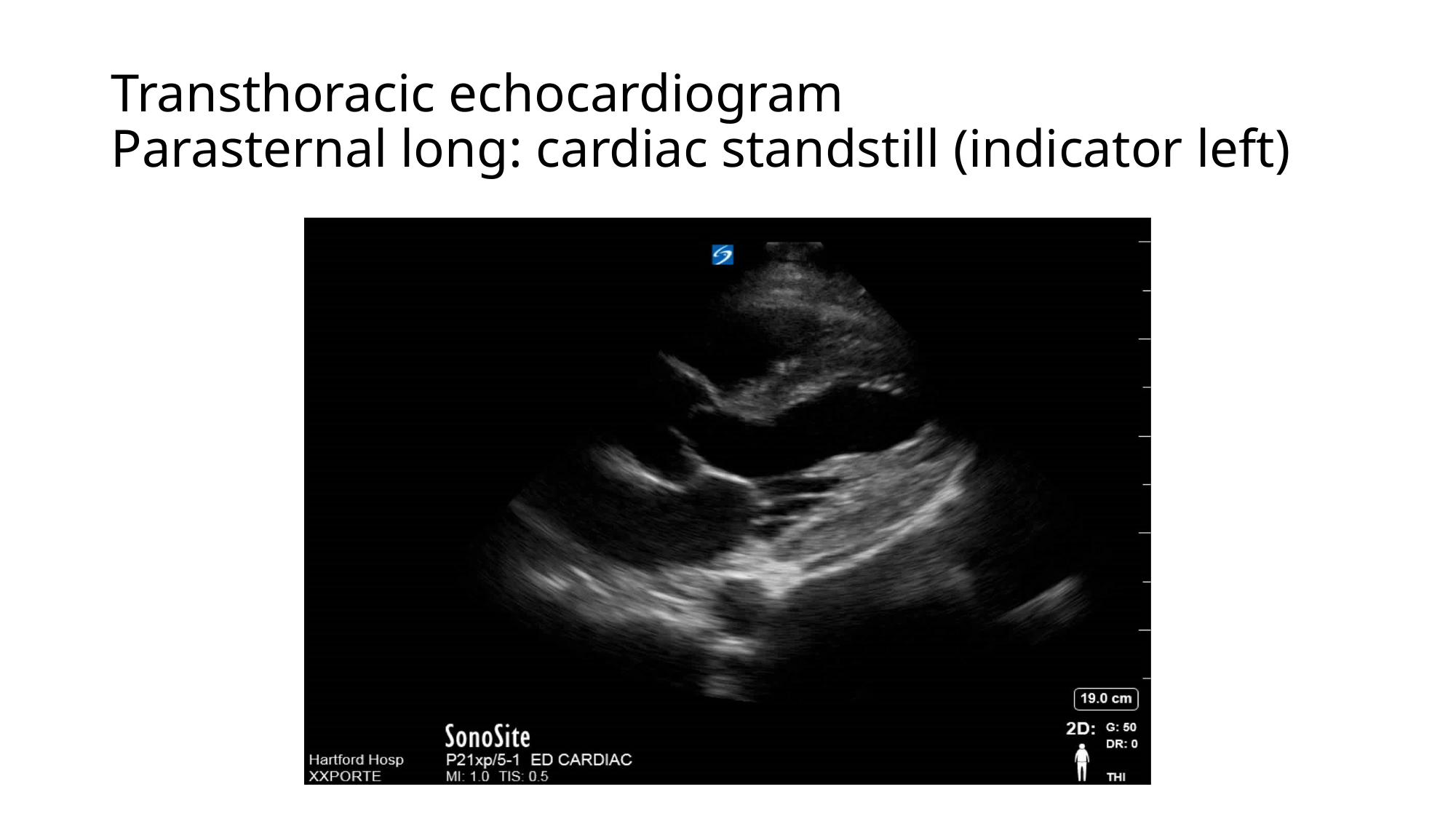

# Transthoracic echocardiogram Parasternal long: cardiac standstill (indicator left)

## Slide 7
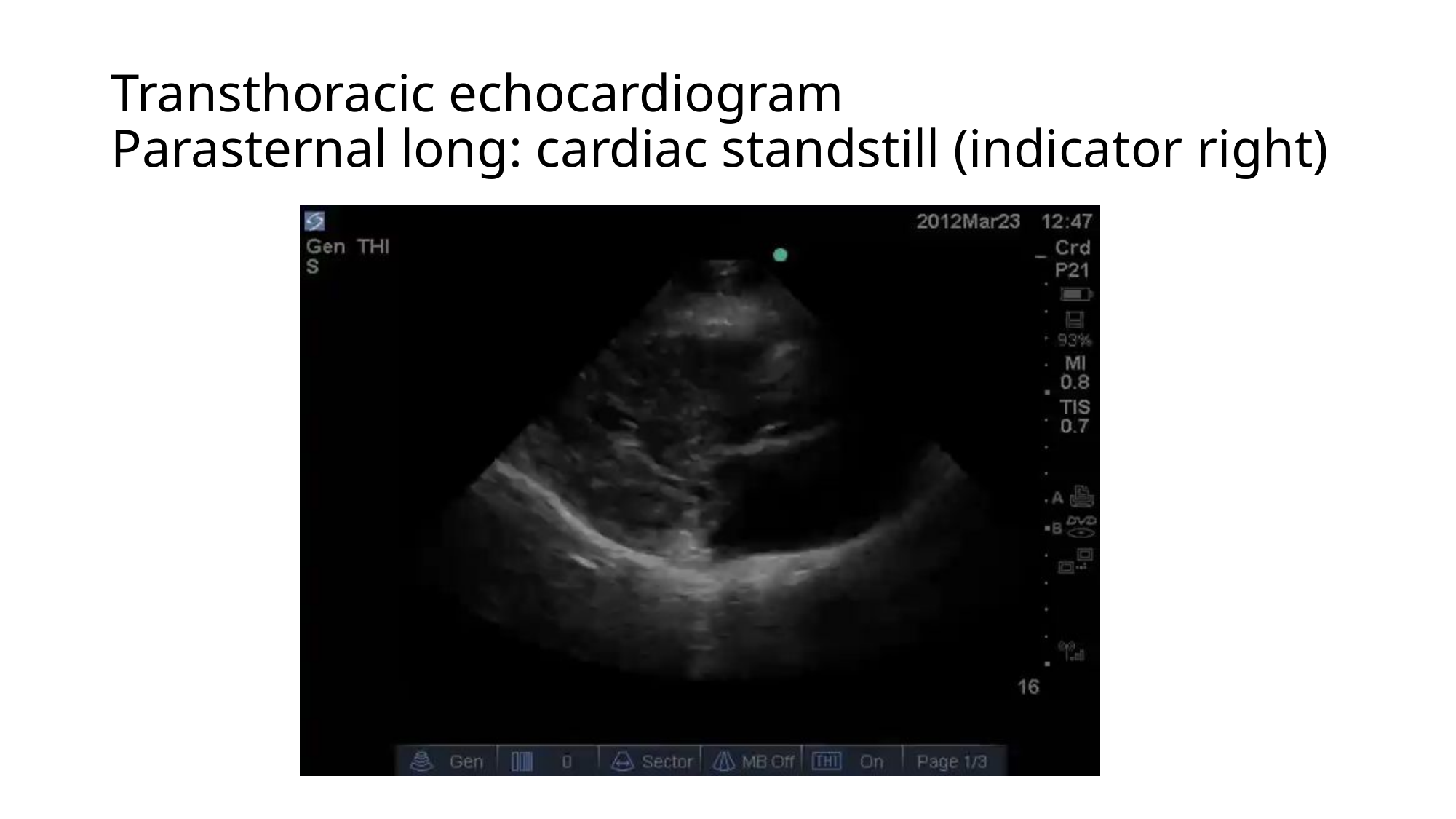

# Transthoracic echocardiogram Parasternal long: cardiac standstill (indicator right)
